# Image reconstruction of fluorescent molecular tomography based on the tree structured Schur complement decomposition

**DOI:** 10.1186/1475-925X-9-20

**Published:** 2010-05-20

**Authors:** Wei Zou, Jiajun Wang, David Dagan Feng

**Affiliations:** 1 School of Electronics and Information Engineering, Soochow University, Suzhou 215021, China; 2 Department of Electronic and Information Engineering, Hong Kong Polytechnic University, Hong Kong; 3 School of Information Technologies, University of Sydney, Sydney, NSW 2006, Australia; 4 Med-X Research Institute, Shanghai Jiao Tong University, Shanghai 200030, China

## Abstract

**Background:**

The inverse problem of fluorescent molecular tomography (FMT) often involves complex large-scale matrix operations, which may lead to unacceptable computational errors and complexity. In this research, a tree structured Schur complement decomposition strategy is proposed to accelerate the reconstruction process and reduce the computational complexity. Additionally, an adaptive regularization scheme is developed to improve the ill-posedness of the inverse problem.

**Methods:**

The global system is decomposed level by level with the Schur complement system along two paths in the tree structure. The resultant subsystems are solved in combination with the biconjugate gradient method. The mesh for the inverse problem is generated incorporating the prior information. During the reconstruction, the regularization parameters are adaptive not only to the spatial variations but also to the variations of the objective function to tackle the ill-posed nature of the inverse problem.

**Results:**

Simulation results demonstrate that the strategy of the tree structured Schur complement decomposition obviously outperforms the previous methods, such as the conventional Conjugate-Gradient (CG) and the Schur CG methods, in both reconstruction accuracy and speed. As compared with the Tikhonov regularization method, the adaptive regularization scheme can significantly improve ill-posedness of the inverse problem.

**Conclusions:**

The methods proposed in this paper can significantly improve the reconstructed image quality of FMT and accelerate the reconstruction process.

## Background

Near-infrared (NIR) light can travel several centimeters through biological tissue, and hence has the potential to qualify the molecular information by fluorochromes in tissue [1]. Recently, there has been increasing interest in the molecularly-based medical imaging method, such as fluorescent molecular tomography (FMT) [2-4], in which the injected fluorophore may accumulate in diseased tissue. During the imaging process, the tissue surface is illuminated with excitation light. Then, the fluorophores are excited to emit the light, which is detected as fluorescence [5]. The process of fluorescent light generation and transportation through tissues can be described by a forward model, so that the surface measurements can be predicted on the basis of a guess of the system parameters and the given source positions. To reconstruct an image, it is necessary to calculate the internal optical and fluorescent properties with the given measured data and sources [6].

One of the major challenges in the reconstruction of FMT is its high computational complexity resulted from extremely large-scale matrix manipulations. Generally, the iterative solution approaches, such as CG method [7] and Gauss-Newton (GN) method [8], are more efficient than the direct solution approaches. Additionally, the iterative methods based on the reduced system can be more efficient than those based on the global system. One of such systems is the Schur complement system, which was firstly used by Haynsworth [9]. The condition number of the Schur complement of a matrix is never greater than that of the given matrix, and hence the convergence properties of iterative solving of linear systems can be significantly improved [7,10]. In this paper, we propose to adapt this idea for the FMT reconstruction. The most important innovation of our method lies in its tree structured level-by-level decomposition strategy, where decompositions in each level are performed in two ways. This strategy is quite different from that in [10] where only one component of the global solution is derived in the Schur complement system. The advantages of our method are obvious because a further improvement in the reconstruction accuracy and speed can be achieved with level-by-level Schur complement decomposition. Another contribution of this paper is that we propose a modified spatially variant regularization method incorporating the objective function to tackle the ill-posed nature of the inverse problem.

## Methods

### Forward Model and Finite Element Formulation

FMT acquisitions are obtained through a two-step image formation model [11]. In the first step, sources at several locations are used to illuminate the tissue. This step, in frequency domain, is driven by the diffusion equation [12](1)

where the subscript *x *denotes the excitation wavelength; ∇ is the gradient operator; *S*_*x*_(W/cm^3^) is the excitation light source; Φ_*x*_(W/cm^2^), *D*_*x*_(cm), and *k*_*x *_(cm^-1^) represent the photon fluence, the diffusion coefficient, and the decay coefficient, respectively; Ω denotes the bounded domain of reconstruction.

In the second step, the fluorophores are excited to emit the fluorescence. The second step can be modelled by a second diffusion equation(2)

where the subscript *m *indicates the emission wavelength, *ω*(*rad/s*) denotes the modulation frequency of the source. *S*_*m *_is the emission light source. The diffusion coefficient *D*_*x,m*_(cm), and the decay coefficient *k*_*x,m *_(cm^-1^) are defined, respectively, as[6](3)(4)

where *μ*_*ax,mi *_(cm^-1^) denotes the absorption coefficient due to endogenous chromophores; *μ*_*ax,mf *_(cm^-1^) represents the absorption coefficient due to exogenous fluorophores;  is the reduced scattering coefficient; *q *is the quantum efficiency of the fluorophore; *τ*(s) is the lifetime of fluorescence; and finally, *c*(cm/s) is the speed of light in the medium.

Here, the Robin-type boundary conditions are implemented on the boundary ∂Ω of domain Ω to solve the above diffusion equations(5)(6)

where **n **is a vector normal to the boundary ∂Ω, *b*_*x,m *_is the Robin boundary coefficient.

To solve the forward problem within the finite element method (FEM) framework, the domain Ω is divided into *P *elements and joined at *N *vertex nodes. The solution Φ_*x,m *_is approximated by the piecewise linear function , with *φ*_*i *_(i = 1...*N*) being basis functions [13]. Hence, equations (1) and (2) can be rewritten as(7)(8)

where(9)(10)

The elements of finite element matrix **A**_*x,m *_can be obtained from the formula(11)

with Ω_*h *_and Γ_*h *_being the bounded domain and its boundary, respectively.

### Inverse Process of FMT

The inverse process of FMT is to estimate the spatial distribution of the optical or fluorescent properties of the tissues from measurements [14]. In the discrete case, the reconstruction problem can be defined as the optimization of the objective function(12)

where *G *is the forward operator, || || is L_2_-norm, **x **and **y **are the calculated optical or fluorescent properties of the tissues and the detector readings, respectively.

Suppose that the objective function *E *attains its extremum at **x **+ *Δ***x**, expanding the gradient of the objective function *E*' about **x **in a Taylor series and keeping up to the first-order term leads to(13)

Equation (13) can be further written as [15](14)

where *T *denotes the transpose, *Δ***y **= **y **- *G*(**x**) is the residual data between the measurements and the predicted data. The Jacobian matrix **J **is a measure of the rate of change in measurement with respect to the optical parameters. It describes the influence of a voxel on a detector reading. **H **is the Hessian matrix, whose entries are the second-order partial derivatives of the function with respect to all unknown parameters describing the local curvature of the function with respect to many variables [16].

Introducing the Tikhonov regularization term to tackle the ill-posedness of the inverse problem and ignoring the Hessian matrix, the solution to the linearized reconstruction problem can be described as follows(15)

where *λ *is a regularization parameter, which can be determined by the Morozov discrepancy principle [17], **I **is an identity matrix.

### Adaptive Regularization Scheme

The problem of image reconstruction for FMT is ill-posed [18]. The Tikhonov regularization technique, as mentioned above, is one of the major methods to reduce the ill-posedness of the problem [19]. However, there exists one protrudent difficulty for this technique in the determination of the regularization parameter. A general unexpected characteristic of the NIR imaging is that the resolution and contrast of the reconstructed images degrade with the increased distance from the sources and the detectors [20]. Considering the fact that the value of the regularization parameter has important effect on the contrast and resolution of the resultant images, one strategy to solve this problem is to use a spatially variant regularization parameter. Meanwhile, it can be inferred that the objective function is related to the regularization parameters [15]. During the process of minimizing the objective function, decreasing *λ *will speed up the convergence if the value of objective function is decreasing, otherwise increasing *λ *can enlarge the searching area (trust-region). Upon the basis of these considerations, we propose a modified regularization method both adaptive to the spatial variations and the objective function.

Suppose that the number of measurements and the number of the vertex nodes are *M *and *N*, respectively. Thus, we have for the matrices in equation (15): *Δ***x**∈**R**^*N *× 1^, *Δ***y**∈**R**^*M *× 1^, **J**∈**R**^*M *× *N*^, and **I**∈**R**^*N *× *N*^. To construct a spatially variant regularization framework, the inverse term of (**J**^*T*^**J **+ *λ***I**)^-1 ^in equation (15) is replaced with (**J**^*T*^**J **+ ***λ***)^-1^, which results in the following equation(16)

where  is a diagonal matrix. Equation (16) can be rewritten as(17)

with *Δx*_*i *_(*i *= 1, 2, ..., *N*) being the component of the vector *Δ***x**. It can be easily seen that each node *p*_*i *_(*i *= 1, 2,...,*N*) in the reconstructed domain is regularized by a corresponding regularization parameter *λ*_*i *_(*i *= 1, 2,...,*N*) respectively. Obviously, the above mentioned Tikhonov regularization can be regarded as a special case of equation (17) when *λ*_1_ = *λ*_2_ = ⋯*λ*_N_ = *λ*.

It was pointed out in [21] that, the resolution and contrast of the images decrease with the increment of the regularization parameters and vice versa. Therefore, the adaptive regularization parameter *λ*_*i *_can be defined as follows to compensate the decrease of the resolution and contrast with the increased distance from the sources and detectors:(18)

where **r**_*i *_is the position of node *p*_*i*_, **r**_*s *_and **r**_*m *_respectively denote the positions of the source and detector closest to the node *p*_*i*_, *c*_1_ and *c*_2_ are two positive parameters determined empirically in our paper.

To make the regularization parameter adaptive to the objective function as defined in equation (12), we propose to incorporate it in the regularization as follows(19)

In equation (19), the arctan function is used to guarantee a relatively small fluctuation range of the regularization parameters and avoid too large values of them. Obviously, regularization parameters determined from equation (19) relate to the objective function in a similar manner to that as pointed out before. In such a way, the regularization parameters are adaptive not only to the spatial variations but also to the variations of the objective function to accelerate the convergence.

### Reconstruction Based on the Schur Complement System

As has been pointed out previously, the iterative methods based on the Schur complement system can be more efficient to solve large-scale problems. Hence, we propose to reconstruct the tomographic image of FMT with level-by-level decomposition in the Schur complement system.

For convenience of discussions, equation (16) can be further rewritten in a more compact form as(20)

where **k **= **J**^*T*^**J **+ **λ **and **b **= **J**^*T *^*Δ***y**.

To solve the inverse problem of FMT in the Schur complement system, the solution space **R**^*n *^is firstly decomposed into two subspaces of U and V with dimensions *m *and *n*-*m*, respectively. Let [**Γ Ψ**] be an orthonormal basis of the solution space **R**^*n*^. The basis of the *m*-dimensional coarse subspace U is formed by the columns of **Γ **∈ **R**^*n *× *m *^and the columns of **Ψ **∈ **R**^*n×(n-m) *^form the basis of the (*n *- *m*) dimensional subspace V.

Therefore, the solution to equation (20) can be expressed with the bases of the two subspaces as follows(21)

where *u *and *v *are the projections of *Δ***x **on the subspaces U and V, respectively.

**Figure 1:**
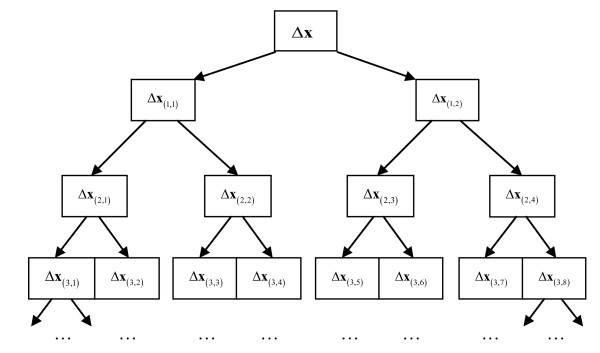
**Schematic illustration of Schur complement decomposition with a tree structure**. The global solution *Δ***x **is decomposed with the Schur complement system level by level along the two paths in the tree structure.

Because both the condition number and the scale of the system can be reduced after Schur complement decomposition, we propose to further decompose both the projections *u *and *v *level by level with the Schur complement decomposition along two paths in a tree structure, and then solve the subsystems in the Schur complement systems. Our approach is different from that proposed in [10], where only the projection *v *is solved in the Schur complement system. The level-by-level Schur complement decomposition can be schematically illustrated as in Figure 1. We derive the iterative system in the following discussions.

Suppose that the subsystem at the *i*th level is as follows(22)

where **S**_(*i*, *j*)_ is the Schur complement matrix with the subscript (*i*, *j*) being the *j*th (*j *= 0, 1,..., 2^*i*^) term at the *i*th (*i *= 0, 1,..., *L*) level in the tree structure as illustrated in Figure 1. Particularly, **S**_(0,0)_ is the global matrix **k **as defined in equation (20). To solve this system in the Schur complement system, equation (22) will be further decomposed at the *i*+1th level. Thus, the solution *Δ***x**_(*i,j*)_ is firstly expressed with the bases of the two subspaces as(23)

where *Δ***x**_(*i*+1,2*j*-1)_ and *Δ***x**_(*i*+1,2*j*)_ are the projections of *Δ***x**_(*i,j*)_ on the subspaces formed by the columns of **Γ**_(*i,j*)_ and **Ψ**_(*i,j*)_, respectively.

Substituting equation (23) into equation (22) yields(24)

Multiplying both sides of equation (24) from the left by [**Γ**_(*i,j*)_**Ψ**_(*i,j*)_]^*T*^, we can obtain(25)

Thus, equation (25) can be further rewritten into a two-by-two block system(26)

where , , , and , while the two components on the right-hand side (RHS) of equation (26) are , and . From equation (26), it can be seen that **S**_(*i,j*)11_ and **S**_(*i,j*)22_ correspond to the equations for the unknowns of *Δ***x**_(*i*+1,2*j*-1)_ and *Δ***x**_(*i*+1,2*j*)_, respectively, while **S**_(*i,j*)12_ and **S**_(*i,j*)21_ define the coupling between these two sets, which will be eliminated in the following discussions.

Applying block Gaussian elimination to equation (26) leads to [22](27)

where , which is called the Schur complement with respect to **S**_(*i,j*)11_[7], . From equation (27), we have(28)(29)

It can be found that the condition number of matrix **S**_(*i*+1,2*j*)_ is smaller than that of matrix **S**_(*i,j*)_[9]. Hence, solving the inverse problem in the Schur complement system at the *i*+1th level will be more efficient than solving it at the *i*th level. We herein solve equation (29) using the biconjugate gradient method [23]. Its advantage is that it does not square the condition number of the original equations [24]. Basically, the biconjugate gradient method can be used to solve the large-scale systems with the fastest speed among all the generalized conjugate gradient methods in many cases [25]. The algorithm for solving equation (29) can be summarized as follows

### Algorithm 1

1. Input an initial guess *Δ***x**_(*i*+1,2*j*)0_;

2. Initialize *d*_0_ = *f*_0_ = *r*_0_ = *p*_0_ ← **b**_(*i*+1,2*j*)_ - **S**_(*i*+1,2*j*)_*Δ***x**_(*i*+1,2*j*)0_;

3. For *k *= 0, 1, 2... until convergence do

   End for

After the derivation of *Δ***x**_(*i*+1,2*j*)_ from equation (29) with algorithm 1, the next task is to obtain the other component of *Δ***x**_(*i*+1,2*j*-1)_ for the synthesis of the solution *Δ***x**_(*i,j*)_. Here, *Δ***x**_(*i*+1,2*j*-1)_ is also solved in the Schur complement system due to its low condition number.

Eliminating the block **S**_(*i,j*)12_ in equation (26) using block Gaussian elimination with **S**_(*i,j*)22_ as pivot block, we have(30)

where  and . Thus **S**_(*i*+1,2*j*-1)_ is the Schur complement with respect to **S**_(*i,j*)22_.

From equation (30), we can obtain(31)

Thus, the solution *Δ***x**_(*i*+1,2*j*-1)_ can be obtained in a same manner as in Algorithm 1, and the only difference is that *Δ***x**_(*i*+1,2*j*)_, **S**_(*i*+1,2*j*)_, and **b**_(*i*+1,2*j*)_ should be replaced with *Δ***x**_(*i*+1,2*j*-1)_, **S**_(*i*+1,2*j*-1)_, and **b**_(*i*+1,2*j*-1)_, respectively. Solving equation (31) is computationally efficient because of the reduced condition number in the Schur complement system [7]. Moreover, such a strategy of deriving both *Δ***x**_(*i*+1,2*j*-1)_ and *Δ***x**_(*i*+1,2*j*)_ in the Schur complement system can be implemented in a parallel manner, since equations (29) and (31) are decoupled. Therefore the subsystem at the *i*th level as in equation (22) can be decomposed into the two linear subsystems at the *i*+1th level, i.e., Schur complement systems as in equations (29) and (31). After obtaining *Δ***x**_(*i*+1,2*j*-1)_ and *Δ***x**_(*i*+1,2*j*)_, they are then substituted into equation (23) to yield the solution *Δ***x**_(*i,j*)_ at the *i*th level. The whole reconstruction algorithm is summarized as follows

### Algorithm 2

1. Set **x**_0_ to an initial guess;

2. **x **← **x**_0_, calculate **b **and **k **at **x **in equation (20) with the adaptive regularization scheme as in equation (19);

3. The global system of equation (20) is decomposed with the Schur complement system level by level in a same manner as the decomposition of equation (22) into equations (29) and (31) to obtain the subsystem **S**_(*i,j*)_*Δ***x**_(*i,j*)_ = **b**_(*i,j*)_ at the *i*th level for *i *=1,..., *L *and *j *=1,..., 2^*i*^, the subspaces at the *i*th level are formed by the columns of **Γ**_(*i,j*)_ and **Ψ**_(*i,j*)_, respectively;

4. Set *i *= *L*;

   For *j *= 1,..., 2^*i *^do

      Combining equations (26), (27), and (30), solve **S**_(*i*+1,2*j*)_*Δ***x**_(*i*+1,2*j*)_ = **b**_(*i*+1,2*j*)_ and **S**_(*i*+1,2*j*-1)_*Δ***x**_(*i*+1,2*j*-1)_ = **b**_(*i*+1,2*j*-1)_ with Algorithm 1, where *Δ***x**_(*i*+1,2*j*-1)_, **S**_(*i*+1,2*j*-1)_, and **b**_(*i*+1,2*j*-1)_ are used instead of *Δ***x**_(*i*+1,2*j*)_, **S**_(*i*+1,2*j*)_, and **b**_(*i*+1,2*j*)_ when Algorithm 1 is employed for the latter case;

   End for

5. While *i *≥ 0

   {

   For *j *= 1,..., 2^*i *^do

      Substitute the solutions *Δ***x**_(*i*+1,2*j*)_ and *Δ***x**_(*i*+1,2*j*-1)_ into equation (23) to obtain the solution *Δ***x**_(*i,j*)_ at the *i*th level;

   End for

   *i *= *i *- 1;

   }

6. **x**_0_ ← **x**_0_ + *Δ***x**_(0,1)_;

7. If ||*Δ***x**_(0,1)_|| >*ε*

         go to 2;

   Else

         **x **← **x**_0_, output **x**.

As mentioned before, the Schur complement system has a smaller condition number than that of the system from which it is constructed [7]. As a result, iterative methods based on the Schur complement systems can be more efficient than the methods based on the global matrix as in equation (20) due to its reduced scale and the smaller condition number. Therefore, the proposed algorithm can be expected to be more efficient than the conventional ones, as the results demonstrated in the next section.

## Results and Discussion

**Figure 2:**
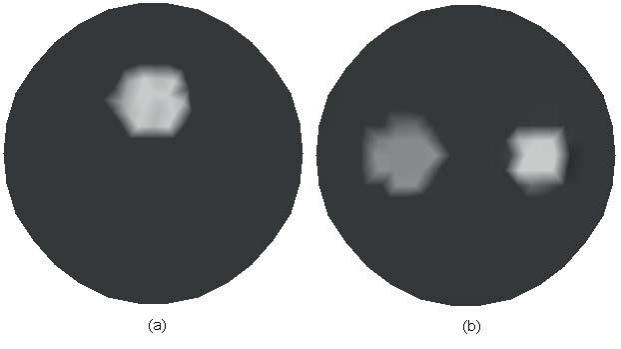
**Simulated phantoms for FMT**. (a) One object with absorption coefficient *μ*_*axf *_of 0.2 *mm*^-1 ^on the background medium with *μ*_*axf *_of 0.06 *mm*^-1^, and (b) two objects of different shapes, one of which with high absorption coefficient of 0.2 *mm*^-1 ^and the other with low absorption coefficient of 0.15 *mm*^-1^, and the background medium with absorption coefficient of 0.06 *mm*^-1^.

**Figure 3:**
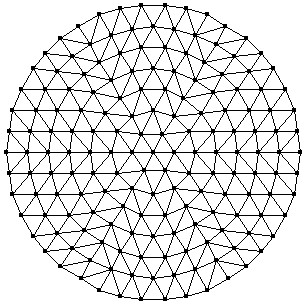
**Mesh used for forward solver of FMT**. The mesh contains 169 nodes and 294 triangular elements.

In this work, assuming that the scattering coefficients are known, we focus on the reconstruction of the absorption coefficient *μ*_*axf*_. Two phantoms as illustrated in Figure 2 are used to evaluate the proposed algorithm. Figure 2(a) contains one object, and Figure 2(b) contains two objects of different shapes. Table 1 and Table 2 outline the optical and fluorescent parameters in different regions of the simulated phantoms corresponding to Figures 2(a) and 2(b), respectively. Four sources and thirty detectors are equally distributed around the circumference of the simulated phantom. The simulated forward data are obtained from equations (1) and (2), in which Gaussian noise with a signal-to-noise ratio of 10dB is added to evaluate the noise robustness of the algorithms. The parameters *c*_1_ and *c*_2_ in equation (19) are, respectively, set to 0.2 and 2. The initial guesses for solutions *Δ***x**_(*i*+1,2*j*)_ and *Δ***x**_(*i*+1,2*j*-1)_ of equations (29) and (31) are set to 0. The initial value of **x**_0_ is set to 5 *mm*^-1^. The subspace created from the right singular vectors of the singular value decomposition (SVD) is optimal. Since SVD is computationally expensive, it is expected that a subspace close to SVD subspace will do almost as good. Thus, the choice of an oscillatory basis can be a basis created by sine or cosine functions with increasing frequency [26]. Here discrete cosine basis is employed in the simulations. To reliably evaluate the performance of different methods for the inverse problem, the best way is to use an independent forward model, which is different from the one employed in the inverse problem, to generate the synthetic data [27]. Therefore, in our case, a finer mesh as shown in Figure 3 with 169 nodes and 294 triangular elements is used to generate the forward simulated data.

**Table 1:** Optical and fluorescent properties of one-object phantom

Excitation light	*μ*_*axf*_(*mm*^-1^)	*μ*_*axi*_(*mm*^-1^)		*q*	*τ*(*ns*)
Background	0.06	0.02	5.0	0.3	0.5
Object	0.2	0.02	5.0	0.3	0.5

Emission light	*μ*_*amf*_(*mm*^-1^)	*μ*_*ami*_(*mm*^-1^)		*q*	*τ*(*ns*)

Background	0.006	0.01	2.0	0.3	0.5
Object	0.1	0.01	2.0	0.3	0.5

**Table 2:** Optical and fluorescent properties of two-object phantom

Excitation light	*μ*_*axf*_(*mm*^-1^)	*μ*_*axi*_(*mm*^-1^)		*q*	*τ*(*ns*)
Background	0.06	0.02	5.0	0.3	0.5
Objects	0.15, 0.2	0.02	5.0	0.3	0.5

Emission light	*μ*_*amf*_(*mm*^-1^)	*μ*_*ami*_(*mm*^-1^)		*q*	*τ*(*ns*)

Background	0.002	0.01	2.0	0.3	0.5
Objects	0.03, 0.05	0.01	2.0	0.3	0.5

**Figure 4:**
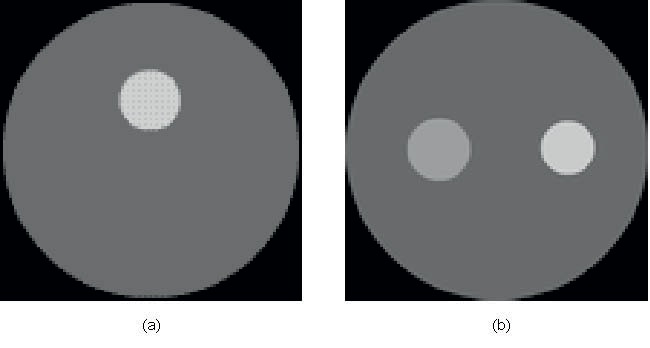
**Model of prior image**. (a) The prior image corresponds to Figure 2(a), and (b) the prior image corresponds to Figure 2(b).

**Figure 5:**
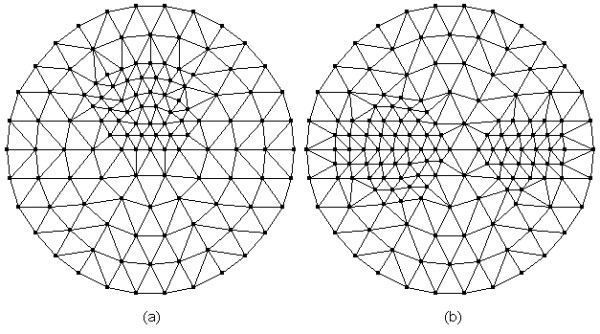
**Meshes used for inverse problem of FMT**. (a) Mesh with 122 nodes and 212 triangular elements, and (b) mesh with 148 nodes and 264 triangular elements..

It is well known that the most significant superiority of the anatomical imaging modality lies in the high spatial resolution. Hence, it will be helpful to improve the image quality and accelerate the reconstruction process if we use the anatomical image as prior information for mesh generation. The reconstructed domain is firstly uniformly discretized according to the Delaunay triangulation scheme, after which the uniform mesh is refined only for the areas with large variations of the pixel values. To simulate this idea, we employ the images shown in Figures 4(a) and 4(b) with a resolution of 100 × 100 pixels as the prior images corresponding to Figures 2(a) and 2(b), respectively. The meshes are generated as shown in Figure 5 for the inverse problem of FMT. The mesh with 122 nodes and 212 triangular elements (Figure 5(a)), and the mesh with 148 nodes and 264 triangular elements (Figure 5(b)) are generated incorporating the prior information as shown in Figures 4(a) and 4(b), respectively.

**Figure 6:**
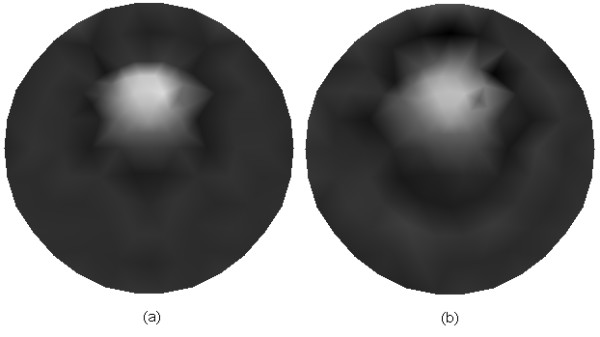
**Reconstructed images of absorption coefficient due to exogenous fluorophores *μ*_*axf *_for one object**. (a) Reconstructed result with the adaptive regularization scheme, and (b) reconstructed result with the Tikhonov regularization method.

**Figure 7:**
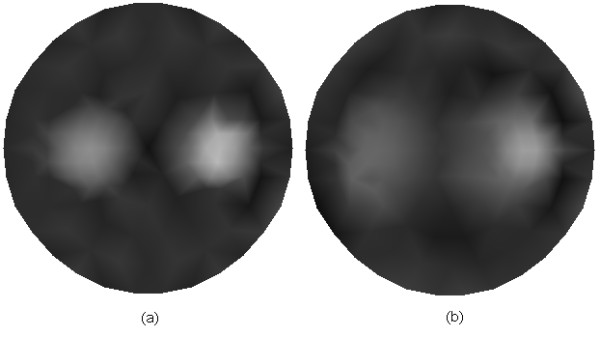
**Reconstructed images of absorption coefficient due to exogenous fluorophores *μ*_*axf *_for two objects**. (a) Reconstructed result with the adaptive regularization scheme, and (b) reconstructed result with the Tikhonov regularization method.

Figures 6(a) and 6(b) show, respectively, the reconstructed images of *μ*_*axf *_for one object phantom using the adaptive regularization scheme and Tikhonov regularization method. Figures 7(a) and 7(b) depict the results for two objects phantom from the above two different algorithms. Here, both of the results from Figures 6 and 7 are based on the CG method. As seen from Figures 6 and 7, better reconstructed results can be achieved from the adaptive regularization scheme. To quantitatively assess the accuracy of the different algorithms, the mean square error (MSE) is introduced(32)

where *N *is the total number of nodes in the domain. The superscript *calc *denotes the values obtained using reconstruction algorithms; and *actual *denotes the actual distribution of *μ*_*axf *_which is used to generate the synthetic image data set. Table 3 lists the performance of the reconstruction algorithms in terms of MSE. It can be seen that the adaptive regularization scheme can significantly improve the quality of the reconstructed images and achieve a smaller MSE in either case.

**Table 3:** Comparison of performance of methods

Methods	One object	Two objects
Adaptive regularization scheme	2.973 × 10^-4^	2.860 × 10^-4^
Tikhonov regularization method	5.352 × 10^-4^	4.892 × 10^-4^

**Figure 8:**
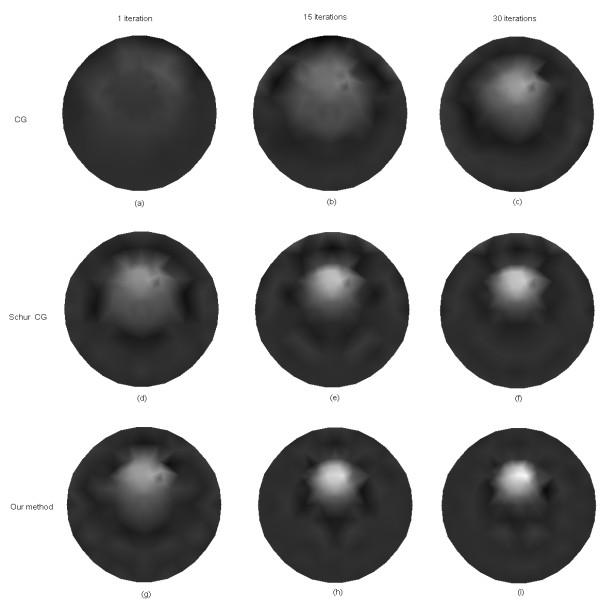
**Reconstructed images of absorption coefficient due to exogenous fluorophores *μ*_*axf *_for one object**. (a)-(c) with the CG method, (d)-(f) with the Schur CG method, and (g)-(i) with our method. The reconstruction results after one iteration are shown in the first column, 15 iterations in the second column, and 30 iterations in the third column.

**Figure 9:**
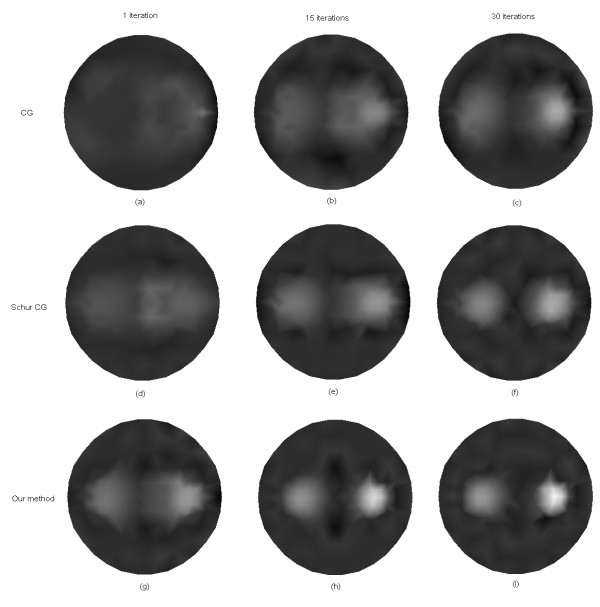
**Reconstructed images of absorption coefficient due to exogenous fluorophores *μ*_*axf *_for two objects**.(a)-(c) with the CG method, (d)-(f) with the Schur CG method, and (g)-(i) with our method. The reconstruction results after one iteration are shown in the first column, 15 iterations in the second column, and 30 iterations in the third column.

Figure 8 shows the reconstructed images of *μ*_*axf *_for one object phantom using the different algorithms after 1, 15, and 30 iterations, respectively. After 30 iterations, the reconstructed image from the proposed algorithm has a relatively higher contrast than those obtained from the other two algorithms. Figure 9 depicts the reconstructed images of *μ*_*axf *_for two objects phantom using the different algorithms, from which it can be seen that the proposed method can reconstruct the images more accurately than the other two methods even after the first iteration. According to the third column of Figure 9, the reconstructed image quality based on our algorithm is significantly improved as compared with that based on the other two methods.

**Figure 10:**
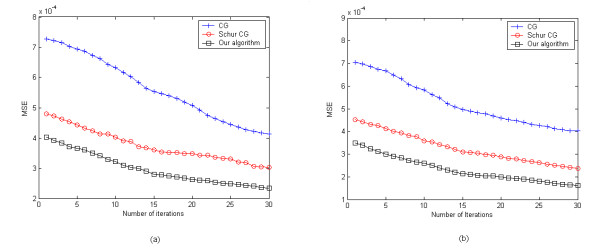
**Number of iterations versus MSE between the original and the reconstructed images**. (a) Reconstruction of one object, and (b) reconstruction of two objects.

We investigated how the MSE changed against the number of iterations for different algorithms. Figure 10 shows a fast convergence of our algorithm with a less MSE than the other two algorithms. In addition, the CG method converges slower than the Schur CG method and our method, which means that solving the inverse problem based on the Schur complement system is superior to that based on the global system. The computation time of different algorithms is further investigated in our work to evaluate the convergence rate. Table 4 lists the computation time after 30 iterations for different algorithms. From this table, it can be seen that the time needed for our algorithm after 30 iterations is less than that of the Schur CG method. Although the former is a little bit longer than the time needed for the CG method, our algorithm needs only less than 5 iterations to achieve the precision of the CG method after 30 iterations. As a result, the CG method needs much more iterations to achieve a given precision of reconstruction than our method. Therefore, compared with the other two methods, the proposed algorithm is more efficient and stable.

**Table 4:** Computation time of FMT image reconstruction for 30 Iterations

Methods	One object	Two objects
CG	62s	86s
Schur CG	203s	281s
Our algorithm	141s	179s

**Figure 11:**
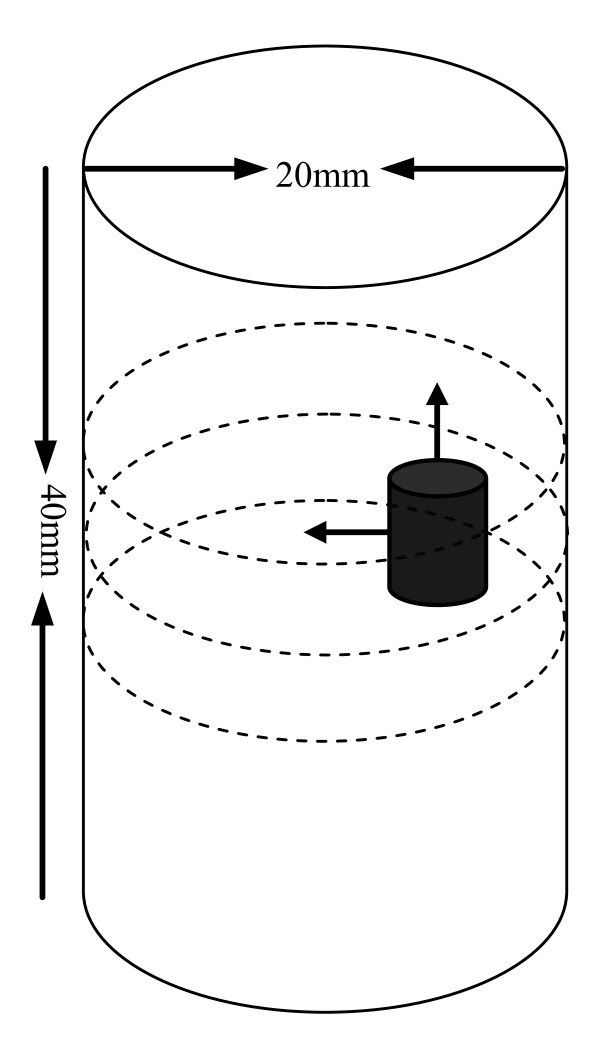
**Simulated phantom for 3D reconstruction**. The phantom of radius 10 *mm *and height 40 *mm *with a uniform background of *μ*_*axf *_= 0.005*mm*^-1 ^, which is positioned at *x *= 10*mm*, *y *= 0*mm *and *z *= 20*mm*. The small cylindrical anomaly has a radius of 2 *mm *and height 6 *mm *with *μ*_*axf *_= 0.01*mm*^-1^. The anomaly is positioned at *x *= 15*mm*, *y *= 0*mm *and *z *= 20*mm*. The dashed curves represent the measurement planes, at *z *= 15*mm*, *z *= 20*mm*, *z *= 25*mm*.

**Figure 12:**
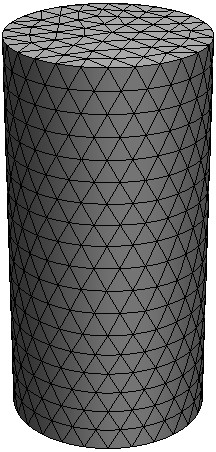
**3D mesh for image reconstruction**. 3D mesh for image reconstruction with 858 nodes and 3208 tetrahedral elements.

**Figure 13:**
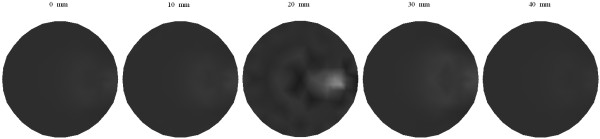
**Reconstructed images using the Schur CG method**. Reconstructed images using the Schur CG method, which are 2D cross sections through the reconstructed 3D volume. The right-hand side corresponds to the top of the cylinder (*z *= 40 *mm*), and the left corresponds to the bottom of the cylinder (*z *= 0 *mm*), with each slice representing a 10 *mm *increment.

**Figure 14:**
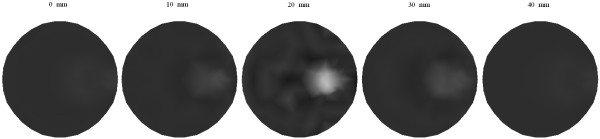
**Reconstructed images using the proposed algorithm**. Reconstructed images using the proposed algorithm, which are 2D cross sections through the reconstructed 3D volume. The right-hand side corresponds to the top of the cylinder (*z *= 40 *mm*), and the left corresponds to the bottom of the cylinder (*z *= 0 *mm*), with each slice representing a 10 *mm *increment.

To further validate the proposed algorithm for 3D reconstruction, a phantom as illustrated in Figure 11 is used for simulations. Within this phantom, a small cylindrical object is suspended. In Figure 11, the dashed curves represent the planes of measurements. Four sources and sixteen measurements are used for each plane in the simulations. The mesh for reconstructing the 3D image is shown in Figure 12, which contains 858 nodes and 3208 tetrahedral elements. Figures 13 and 14 depict the reconstructed 2D cross sections of the 3D phantom shown in Figure 11 using the Schur CG method and the proposed algorithm, respectively. Table 5 lists the performance of the above two methods for a quantitative comparison. From this table, we can conclude that our proposed algorithm can also speed up the reconstruction process and achieve high accuracy for the 3D case.

**Table 5:** Performance comparison of reconstruction methods for 3D case

Methods	Schur CG	Our algorithm
Computation time (s)	3527	2215
MSE	3.629 × 10^-3^	1.241 × 10^-3^

## Conclusion

In this paper, we developed a novel image reconstruction method of FMT, based on the tree structured Schur complement decomposition in combination with the adaptive regularization scheme. The proposed approach decomposes the global inverse problem level by level with the Schur complement decomposition, and the resultant subsystems are solved with the biconjugate gradient method. The spatially variant regularization parameter is determined adaptively according to the objective function. Simulation results demonstrate that the proposed method outperforms the previous methods, such as the CG and the Schur CG methods, in both reconstruction accuracy and speed.

## Competing interests

The authors declare that they have no competing interests.

## Authors' contributions

WZ conceived the study, implemented the algorithm, and drafted the initial manuscript. JJW participated in the design of the study, analyzed the simulation results, and helped to draft the manuscript. DDF critically reviewed the manuscript, helped to analyze the results, revised the manuscript, and provided the valuable advice.
